# Advancing evaluation of AI systems when humans make the decisions

**DOI:** 10.1073/pnas.2523997122

**Published:** 2025-12-15

**Authors:** Alexander D’Amour

**Affiliations:** ^a^Google DeepMind, Cambridge, MA 02142

AI systems have become increasingly flexible, making it feasible to integrate them into a wide range of human decision-making processes, from criminal justice (1), healthcare (2), and financial services (3) to personal administrative tasks. As a result, questions about how AI systems can improve or automate human decision-making are on the rise. These decisions can have high stakes, which makes careful evaluation of new AI-assisted or automated systems essential. However, these stakes also complicate evaluation: How can we evaluate unproven AI systems in domains where we currently only trust humans to make decisions?

Ben-Michael et al. (4) develop a formalism and suite of methods for this setting. The core problem they address is that the data we use for evaluation were collected under the very decision system we seek to improve. This is known as the selective labels problem (5). Ben-Michael et al (4) clarify and improve upon heuristics that are currently used to evaluate systems under selective labels. In addition, they develop rigorous, statistically efficient methodology for estimating key metrics, and demonstrate the methodology in a real high-stakes criminal justice setting.

For this comprehensive treatment, Ben-Michael et al. (4) should be required reading for those tasked with evaluating emerging AI-assisted and automated decision systems. In this comment, I will elucidate some of the key conceptual points of the paper, with an eye toward practitioners who are more familiar with evaluating automated systems in AB testing or offline evaluation settings (6, 7).

Ben-Michael et al. (4) discuss their framework in the context of applications of AI to bail decisions in criminal justice, but the selective labels problem is ubiquitous. I had my own experience with selective labels as a graduate student, when I consulted for a company that was exploring how machine learning methods, which we might now brand as AI, could improve and scale microlending decisions. The “live” system, from which we drew our data, comprised a set of human loan officers, who decided on loan applications. In cases where loans were approved, repayment data were recorded. When evaluating an AI system using this dataset, it was easy to evaluate cases where the AI system was more stringent than human loan officers. In these cases, a human loan officer opted to approve a loan, but the AI system (retrospectively) predicted the applicant would default; when this occurred, we had repayment data, and we could directly observe in repayment data whether the borrower would default, as the AI system predicted. However, in the opposite case, we were unable to observe what would have happened in cases where the AI system recommended approving a loan, but the human loan officer did not. In these cases, there was no actual loan, and thus no repayment data. This made it difficult to measure how implementing an AI-assisted or automated system might navigate the tradeoffs of expanding access to credit against solvency of the lender. I will use this as a running example.

There are two core ideas in Ben-Michael et al.’s treatment of this problem that may be less familiar to many practitioners. The first idea draws a distinction between two kinds of decision systems: human-involved systems (in which a human ultimately makes the decision) and AI-alone systems. These systems differ on three key properties: first, whether they are live or offline; second, whether they are backtestable; and third, whether we can account for all of their inputs. The human-involved systems are “live,” in the sense that their decisions are actually applied in the world, and yield observations of outcomes (like repayment data). However, the decisions from these systems cannot be backtested, or applied retrospectively on previously decided cases. This means, for example, that for a decision that was actually made by a loan officer with AI assistance, we cannot simultaneously observe how that officer would have decided without AI assistance. Finally, the human part of human-involved systems may incorporate “intangibles” that are not recorded, resulting in unobserved confounding that prevents unbiased imputation of unseen outcomes. By contrast, the AI-alone systems are “offline,” in that their decisions are not applied to the world, but they can be backtested. Further, we usually know exactly which inputs are given to the AI-alone system. Thus, for some decisions made by the AI system (e.g., extending a loan to a given applicant), we can only observe its consequences when a live system made the same decision. However, for any case that was actually decided by a live system, we can simultaneously observe what the AI system would have recommended, and know what inputs went into that decision.

The second conceptual contribution of the paper states the evaluation goal in terms of potential outcomes. Formally, for a system tasked with making a binary decision d for units indexed by i, the authors use $Y_{i}\left(d\right)$ to denote the outcome that we would have observed had decision d been made for unit i. In my lending example, the lender decides whether to extend a loan (d=0), or to deny the loan application (d=1). (Here, the convention is that the “favorable” decision value d takes the value 0.)

In the paper’s notation, the potential outcome of interest for each unit is denoted $Y_{i}\left(0\right)$. In the lending example, $Y_{i}\left(0\right)=1$ if the borrower defaults on their loan, and is 0 if the borrower repays. (The potential outcome $Y_{i}\left(1\right)$, the outcome when the loan is denied, is defined to have a known deterministic value, and is ignored.) Given this setup, Ben-Michael et al. state the goal as evaluating how well different systems classify units according to $Y_{i}\left(0\right)$. The difficulty is that, from the live system that generated the data, the decision is not 0 for all units, so $Y_{i}\left(0\right)$ is not observed for all units. Ben-Michael et al. consider how to measure improvements in classifying $Y_{i}\left(0\right)$ with minimal assumptions about unobserved outcomes. In particular, the central condition is embedded in the potential outcomes notation: The outcome $Y_{i}\left(d\right)$ for unit i depends only on the decision that was applied to that unit, not on *how* that decision was made (whether human-alone, AI-assisted, or AI-alone; this is the “single-blind” assumption), or on the decisions made for other units (i.e., no interference).

With this setup, the core theoretical results in the paper establish how well we can measure classification performance of live and offline decision systems under selective labels. The authors focus on the case where we compare two live, human-involved systems in a randomized AB-test-like trial (or an observational study that can be adjusted to mimic one), where one arm is AI-assisted and the other is not. This is a clever choice because it enables a view into the impacts of AI systems while ensuring that a human expert is still making the live high-stakes decisions. There are two key results, one concerning the difference in classification performance between the two live human-involved systems in the trial (Theorem 1), and the other concerning the performance of an offline AI-only system (Theorem 3). I will consider each of these results in turn.

In Theorem 1, Ben-Michael et al. show that the difference in classification ability between the two live human-involved systems in the trial can be identified based on observables alone, despite partial observability of Y(0). This raises the question of how an evaluation based on this classification risk difference compares to current evaluation heuristics. For the latter, consider the problem framing in ref. 5, the foundational work that coined the “selective labels” term. Lakkaraju et al. also formulate the problem in terms of partially observed outcomes, but propose comparing algorithms on two observable dimensions assumed to trade off: their “acceptance rate,” or the rate at which a decision was made such that consequences could be observed ($\text{Pr}(D^{*}=0)$ for some decision process $D^{*}$ in the notation of Ben-Michael et al. (4)), and their “failure rate,” or the rate at which poor outcomes occurred across the entire population ($\text{Pr}(D^{*}=0,Y\left(0\right)=1)$ in the current notation).

As it turns out, it follows quite straightforwardly from Theorem 1 that the risk difference with respect to Y(0) corresponds to a linear combination of the differences in the acceptance rate and failure rate metrics. Recall that Ben-Michael et al. use Z to denote whether AI assistance is assigned, D(Z) the decision made had AI assistance status Z been assigned, and $p_{\mathrm{yd}}\left(D\left(\cdot \right)\right)=\;\text{Pr}\left(Y\left(0\right)=y,D\left(\cdot \right)=d\right)$ the joint probability of potential outcome y co-occurring with decision d. In the simple case where we do not observe covariates X, and AI assistance Z is assigned completely at random, the expression in Theorem 1 can be written

$$\begin{array}{cc} R_{\text{HUMAN+AI}}\left(l_{01}\right)-R_{\text{HUMAN}}\left(l_{10}\right) & \\ & =p_{10}\left(D\left(1\right)\right)-p_{10}\left(D\left(0\right)\right)+l_{01}\left(p_{01}\left(D\left(1\right)\right)-p_{01}\left(D\left(0\right)\right)\right)\\ & =p_{10}\left(D\left(1\right)\right)-p_{10}\left(D\left(0\right)\right)+l_{01}\left(p_{00}\left(D\left(1\right)\right)-p_{00}\left(D\left(0\right)\right)\right)\\ & \begin{array}{l} =p_{10}\left(D\left(1\right)\right)-p_{10}\left(D\left(0\right)\right)\\ \;\;\;\;\;\;\;\;\;\;l_{01}\{\left[\mathrm{Pr}\left(D\left(1\right)=1\right)-p_{10}\left(D\left(1\right)\right)\right]\\ \;\;\;\;\;\;\;\;\;\;\;\;\;\;\;\;\;\;\;\;\;\;\;\;-\left[\mathrm{Pr}\left(D\left(0\right)=1\right)-p_{10}\left(D\left(0\right)\right)\right]\}\\ =\left(1+l_{01}\right)\;\;\underset{\text{Difference in false negative proportion}}{\underset{︸}{\left[p_{10}\left(D\left(1\right)\right)-p_{10}\left(D\left(0\right)\right)\right]}}\\ \;\;\;\;\;\;\;\;\;\;\;\;\;-l_{01}\underset{\text{Difference in "acceptance" rate}}{\underset{︸}{\left[\mathrm{Pr}\left(D\left(1\right)=1\right)-\mathrm{Pr}\left(D\left(0\right)=1\right)\right]}} \end{array} \end{array}$$

This result shows that practitioners following current heuristics in a randomized trial (or AB test) of human-involved systems need not make drastic changes to incorporate the paper’s insights. However, the formulation in terms of potential outcomes is still valuable. It suggests a principled way of navigating the tradeoff between acceptance rate and failure rate in terms of the relative costs of false positives and false negatives with respect to Y(0). This can be a particularly useful translation when the costs of false positives and false negatives accrue to different parties. For example, in the lending example, observable false negative errors most directly affect the lender’s bottom line, while unobservable false positive errors most directly affect the applicant. Reasoning about the tradeoffs of these costs explicitly can give a qualitatively different picture from formulating the problem in terms of the precision of lending decisions and loan acceptance rates.

Turning to Theorem 3, Ben-Michael et al. show that the difference in classification quality between a live human-involved system and an offline AI system cannot be identified exactly, but provide sharp bounds on the difference. Interestingly, Theorem 3 shows that data from a randomized trial comparing live human-involved systems can be used to bound an offline AI system’s performance more sharply than comparing the AI system to each human-involved system in isolation. To see this, we can rewrite their bound in the case where there is only one live system. Here, the authors introduce A, the decision recommended by an offline AI system. Considering the case of no covariates, the gap between the upper bound U and the lower bound L on the difference in classification risk between the live system and the offline system is

$$\begin{array}{cc} U-L= & \left(1+\ell_{01}\right)[\;\text{Pr}\left(A=0\right)\\ & -\;\text{Pr}(Y=1,D=0,A=0)\\ & -\;\text{Pr}(Y=0,D=0,A=0)]\\ & =\left(1+\ell_{01}\right)\left[\;\text{Pr}\left(A=0\right)-\;\text{Pr}\left(D=0,A=0\right)\right]\\ & =\left(1+\ell_{01}\right)\;\text{Pr}\left(D=1,A=0\right) \end{array}$$

This bound width results from the offline nature of the AI system A. Specifically, the units for which $D_{i}=1$ and $A_{i}=0$ are an ambiguous set. In the lending example, these are the units to which human loan officers denied a loan, but which the AI system would have accepted. For these units, we do not know whether extending a loan under the AI-only decision A would have resulted in default or repayment. The bound width results from assuming that either all units in this subpopulation have a negative outcome $Y_{i}\left(0\right)=1$ (e.g., all “new” units recommended by the AI-only system default, resulting in higher risk for the AI-only system) or a positive outcome $Y_{i}\left(0\right)=0$ (e.g., all “new” units repay, resulting in lower risk for the AI-only system).

For this comprehensive treatment, Ben-Michael et al. (4) should be required reading for those tasked with evaluating emerging AI-assisted and automated decision systems.

**Fig. 1.**
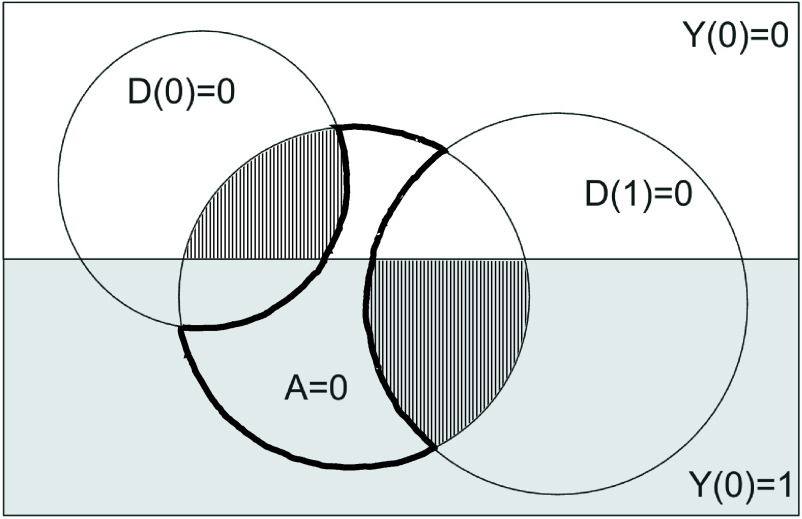
Combining selectively labeled data from a randomized trial of two live human-involved systems D(z), z∈{0,1}, yields sharper bounds on AI-alone system performance A than comparing to either system in isolation. The Venn diagram represents the whole population by the large rectangle, split by the value of Y(0) into the *Upper* and *Lower* regions. Outcomes Y(0) are only observable for subpopulations where D(z)=0. Ideally, only outcomes in the heavily outlined region would be ambiguous; however, the level of overlap between the D(1)=0 and D(0)=0 subpopulations is also ambiguous. (They are shown as disjoint here for visual clarity.) The optimal bound on the classification risk of A relative to D(0) and D(1) simultaneously uses observed outcomes from the vertically shaded subpopulations, which can include observations revealed by two separate live decisions systems D(z).

Theorem 3 shows how much better we can do by pooling information from both arms of the live randomized trial to reduce the size of this ambiguous set. The strategy, which cannot be improved without further assumptions about the live systems, is illustrated in Fig. 1. Suppose the diagram represents a randomized trial between two live loan approval policies (say, loan officers following different rubrics), D(0) and D(1). Here, D(0) is less permissive (the circle representing units for which $D_{i}\left(0\right)=0$ is smaller), and more precise (the D(0)=0 circle mostly overlaps with the Y(0)=0 part of the population); meanwhile, D(1) is more permissive, but less precise.

As before, when making a comparison to an offline AI system A, there will be an ambiguous set of units for which the difference in outcomes between the live and offline system is unobservable. Ideally, only the region where A=0 and neither human-involved system decided 0 would be ambiguous (region outlined with the heavy black line in Fig. 1). However, there is a complication: We cannot make any inferences about potential intersections between the regions where (A=0,D(0)=0) and (A=0,D(1)=0); that is, we do not know how often D(1) and D(0) would have approved the same or different loan applicants. This is because we cannot observe the decisions from both human-involved systems for any given unit (recall, they are not backtestable), and further, we cannot make standard confounder adjustments, because the live decisions could depend on unrecorded inputs. In Fig. 1, these regions are shown as disjoint, but there is actually ambiguity about how much they overlap.

Instead, Theorem 3 shows that the best we can do is to choose two disjoint sets of units to treat as unambiguous. In particular, we can separately take the cases where (Y=0,D(z)=0,A=0) for one value of z, and $\left(Y_{i}=1,D\left(z\right)=0,A_{i}=0\right)$ for one value of z, choosing for each set the value of z that maximizes probability mass. (These sets are observably disjoint because they contain units with different observed values of $Y_{i}\left(0\right)$.) These are shown as the regions shaded with vertical lines in Fig. 1. In our example, these regions correspond to the AI-accepted borrowers approved by the precise policy who repaid [the region where (D(0)=0,Y(0)=0,A=0)], and the AI-accepted borrowers approved by the permissive policy D(1) who defaulted [the region where (D(1)=0,Y(0)=1,A=0)]. We cannot know which repayers approved by D(1) would have also been approved by D(0), or which defaulters approved by D(0) would have been included in those approved by D(1), so the remaining units remain ambiguous. The bounds on risk difference then have a width proportional to the probability mass in the A=0 circle with these shaded regions removed. Notably, this results in a tighter bound than the heuristic previously suggested in Lakkaraju et al. (5), which bounds the performance of the offline AI-only system by comparing to a single live system with the highest acceptance rate [in this case, the permissive policy D(1)].

Here, I have focused on population-level results in Ben-Michael et al. (4), which use potential outcomes formalism to precisely characterize what can be learned about online and offline systems under selective labels. However, the primary practical strength of this paper is that it brings modern semiparametrically efficient estimation methodology to bear on this problem. This methodology, which has shown utility in the causal inference field, comes with guarantees of statistical efficiency and the flexibility to apply this approach to studies with more complexity than completely random trials. (These guarantees are subject to some conditions clearly laid out in the paper. For estimating the difference between live systems, they require that nuisance components, corresponding to predicting outcomes and assignment to live treatment arms, can be estimated consistently and at an appropriate rate. These conditions are particularly mild in the case of a randomized trial. For estimating bounds on offline system performance, there is an additional margin condition: It should not be too hard to determine which sets of units to carve out of the ambiguous set in Fig. 1.) Having this methodology clearly laid out from the start, as well as a real applied example from the criminal justice context to work off of, should make this approach immediately actionable. As AI systems are increasingly incorporated into established human decision-making processes, I expect (and hope) that this approach will become a key part of the evaluation toolbox.
